# Identification of enzymes involved in SUMOylation in *Trypanosoma brucei*

**DOI:** 10.1038/srep10097

**Published:** 2015-05-11

**Authors:** Kaiqin Ye, Xuecheng Zhang, Jun Ni, Shanhui Liao, Xiaoming Tu

**Affiliations:** 1 Hefei National Laboratory for Physical Sciences at Microscale, School of Life Sciences, University of Science and Technology of China, Hefei, Anhui 230026, P.R. China; 2 School of Life Sciences, Anhui University, Hefei, Anhui 230039, P.R. China; 3 Anhui Provincial Engineering Technology Research Center of Microorganisms and Biocatalysis, 111 Jiulong Road, Hefei, Anhui 230601, P.R. China

## Abstract

Small ubiquitin-like modifier (SUMO), a reversible post-translational protein modifier, plays important roles in diverse cellular mechanisms. Three enzymes, E1 (activating enzyme), E2 (conjugating enzyme) and E3 (ligase), are involved in SUMO modification. SUMOylation system and process in higher eukaryotes have been well studied. However, in protozoa, such as *Trypanosoma brucei* (*T. brucei*), these remain poorly understood. Herein, we identified the E1 (TbAos1/TbUba2) and E2 (TbUbc9) enzymes of SUMOylation pathway in *T. brucei* by sequence analysis and GST pull-down assay. Furthermore, we successfully reconstructed the SUMOylation system *in vitro* with recombinant enzymes. Using this system, the active site of TbUba2 and TbUbc9 was revealed to be located at Cys343 and Cys132, respectively, and a centrin homologue (TbCentrin3) was identified to be a target of SUMOylation in *T. brucei*. Altogether, our results demonstrate that TbAos1/TbUba2 and TbUbc9 are the *bona fide* E1 and E2 enzymes of the SUMOylation system in *T. brucei*.

Post-translational modifications, such as phosphorylation, acetylation, methylation and ubiquitination, play pivotal roles in a number of cellular processes including protein quality control, protein localization, signal transduction, DNA damage response and so on^1^,^2^. SUMO (Small Ubiquitin-related MOdifier) was identified to be a reversible post-translational modifier^3^. SUMO system utilizes a similar modification pathway to that of ubiquitin system, and has been reported to be essential for a variety of cellular processes such as kinetochore assembly, chromosome segregation and transcription regulation^4^,^5^,^6^,^7^.

The SUMOylation process is conserved from yeast to metazoa. Similar to ubiquitination, three enzymes (E1, E2 and E3) are responsible for SUMO conjugation^3^. In brief, at first, SUMO is activated and covalently linked to the heterodimer E1 enzyme (Aos1/Uba2) by forming a thioester bond between SUMO C-terminal carboxyl group of GG motif and the cysteine residue of Uba2 in the presence of ATP^8^,^9^,^10^,^11^. SUMO is then transferred from Uba2 to E2 conjugating enzyme (Ubc9), forming a thioester linkage similar to the SUMO activating step^12^,^13^,^14^. Finally, SUMO is conjugated to the substrate in the presence or absence of E3 ligase. In human genome, four SUMO family members (SUMO1, SUMO2, SUMO3 and SUMO4) were identified, which can be targeted to different substrates and participate in different cellular processes^15^,^16^,^17^,^18^,^19^. However, many unicellular organisms harbor only one SUMO homologue to maintain cell life.

*Trypanosoma brucei* is a parasitic protozoa, which causes African sleeping sickness in sub-Africa region. It has been reported that there is only one SUMO homologue (TbSUMO) in *T. brucei*. In our previous studies, TbSUMO was found to be located dominantly in the nucleus and involved in the regulation of chromosome segregation in *T. brucei*^20^. Here, we employed *in silico* method to search the homologues of E1 and E2 in *T. brucei* genome, and confirmed the identities of the E1 (TbAos1/TbUba2, Tb11.02.5410/Tb927.5.3430) and E2 (TbUbc9, Tb927.2.2460) by pull-down and *in vitro* SUMOylation assays. Furthermore, we revealed the active sites of TbUba2 and TbUbc9 and identified a conserved centrin protein as a target of SUMOylation.

## Results

### Identification of E1 and E2 homologues of the SUMOylation system in *T. brucei*

**Figure 1:**
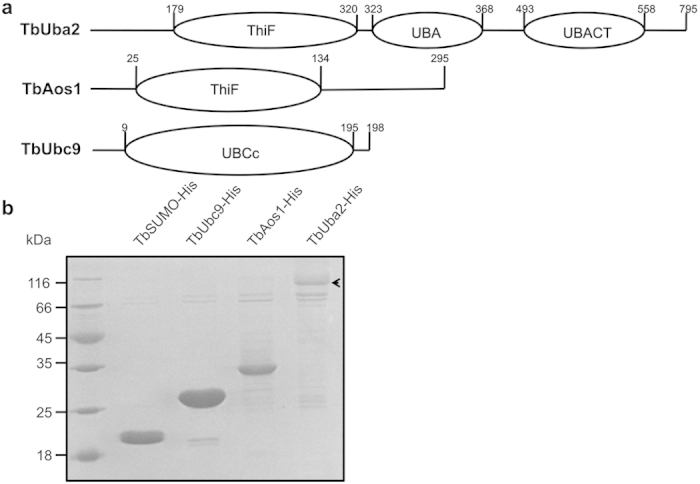
(**a**) Domain architectures of TbUba2, TbAos1 and TbUbc9. The primary sequences of the proteins were analyzed by SMART (http://smart.embl-heidelberg.de). (**b**) SDS-PAGE analysis (Coomassie blue stained) of purified TbSUMO-His, TbAos1-His, TbUba2-His and TbUbc9-His. TbUba2 is indicated with arrow.

It has been shown earlier that the sequence of activating enzyme E1 displays weak but significant similarity to the sequence of MoeB from *E. coli*^21^. In addition, since the discovery of Urm1, an ubiquitin-like protein from *Saccharomyces cerevisiae*, a number of lines of evidence indicated that the Ubl-conjugating system may be evolved from sulfur-transfer system of prokaryote^22^. Thus, activating enzyme E1 should have evolved from MoeB/ThiF homologues, and share similar domains with MoeB/ThiF. However, for some reason, the E1 enzyme involved in SUMOylation was splited into two subunits (Aos1 and Uba2) during the evolution. In order to identify the E1 enzyme of *T. brucei*, we employed Aos1 and Uba2 from other organisms as baits to search potential homologues of Aos1 and Uba2 against the genome database of TriTrypDB (http://tritrypdb.org/tritrypdb/). Two protein candidates (Tb11.02.5410-TbAos1 and Tb927.5.3430-TbUba2) were recognized as the potential subunits of E1 of *T. brucei*. The primary sequence of TbAos1 shares about 23% identity and 36% similarity with that of human Aos1, while TbUba2 shares about 24% identity and 37% similarity with that of human Uba2. Additionally, domain analysis (http://smart.embl-heidelberg.de/) suggested that TbAos1 and TbUba2 contain the ThiF domain shared by E1 (Fig. 1a). We exploited the same method to identify the E2 enzyme of *T. brucei*, and recognized Tb927.2.2460 (TbUbc9) as the Ubc9 homologue in *T. brucei*. Sequence alignment indicated TbUbc9 is highly similar to human Ubc9 in sequence, except for an extra loop ranging from Arg31 to Ser71 (see Supplementary Fig. S1 online). For further characterization of the E1 and E2 enzymes, TbAos1, TbUba2, TbUbc9 and TbSUMO were recombinantly expressed in *E. coli* and purified (Fig. 1b).

### Interactions between TbAos1, TbUba2, TbUbc9 and TbSUMO

**Figure 2:**
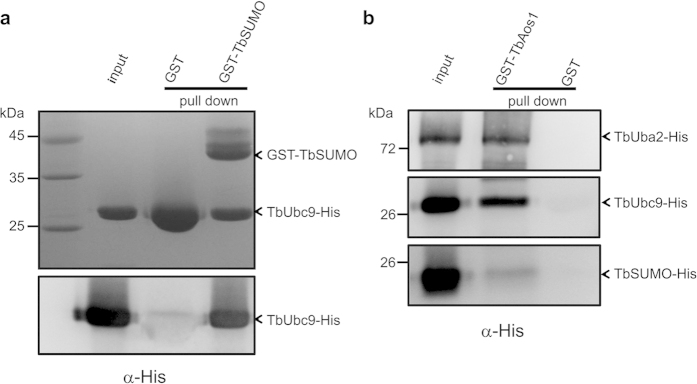
SDS-PAGE (12%) (Coomassie blue stained) and western blotting analysis of GST pull down samples **a**) GST-TbSUMO pull down TbUbc9-His. SDS-PAGE analysis (Coomassie blue stained) of GST-TbSUMO pull down samples (upper). Due to the similar sizes of TbUbc9 and GST, western blotting was performed using anti-His antibody (Lower). GST protein was marked with asterisk (*). (**b**) Western blotting analysis of GST-TbAos1 pull down samples. GST-TbAos1/TbUba2-His (upper), GST-TbAos1/TbUbc9-His (middle) and GST-TbAos1/TbSUMO-His (lower). GST-apo pull down was used as control.

SUMOylation is a well-known ubiquitin-like conjugation process, in which SUMO is activated by enzyme E1, and transferred to enzyme E2, and then ligated to targets with or without the help of enzyme E3^1^. In the SUMO-conjugation pathway, there is a complex interaction network between Aos1, Uba2, Ubc9 and SUMO^23^. Investigation of the interactions between TbAos1, TbUba2, TbUbc9, and TbSUMO would be helpful for us to confirm their identities in the SUMO system of *T. brucei*. Thus, GST pull-down assay was exploited to verify the interactions between these proteins. GST-TbSUMO pull-down result indicated that TbUbc9 associated with TbSUMO tightly. Since TbUbc9 displays high sequence similarity to human Ubc9, the strong interaction between TbSUMO and TbUbc9 indicated TbUbc9 might be the E2 enzyme of SUMOylation system of *T. brucei* (Fig. 2a). Similarly, GST-TbAos1 pull down results showed substantial interactions between TbAos1 and TbUba2 and TbUbc9 (Fig. 2b), implying TbAos1/TbUba2 should be the E1. All these results suggested that TbAos1, TbUba2, and TbUbc9 might be the components of SUMOylation system of *T. brucei*.

### Capability of TbUba2 transferring TbSUMO to TbUbc9

**Figure 3:**
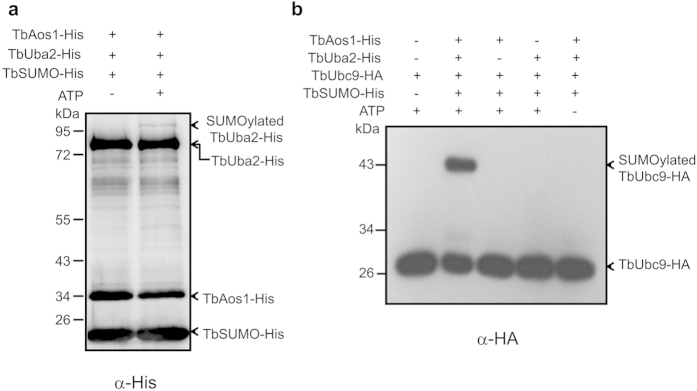
Identification of the enzyme activity of TbUba2. (**a**) *In vitro* SUMOylation of TbUba2 with or without ATP. The reaction was stopped with 2 × SDS-loading buffer. The samples were fractionated by SDS-PAGE (12%) and analyzed by immune blotting using anti-His antibody. The arrows indicate TbSUMO-His, TbAos1-His, TbUba2-His and the SUMOylated TbUba2-His, respectively. (**b**) *In vitro* SUMOylation of TbUbc9-HA in the presence or absence of various components. The samples were fractionated by SDS-PAGE (12%) and analyzed by immune blotting using anti-HA antibody. The arrows indicate TbUbc9-HA and SUMOylated TbUbc9-HA, respectively.

Uba2 interacts with Aos1 to form a heterodimer enzyme E1, which catalyzes SUMO activation in the presence of ATP^8^,^9^,^10^,^11^. In brief, in the presence of ATP, C-terminal carboxyl group of SUMO is adenylated by E1 enzyme, then transfered to a cysteine residue of E2 enzyme to form a thioester bond and store the transfer energy. In order to investigate the enzyme activity of TbUba2, *in vitro* TbUba2-SUMOylation assay was performed. The result showed that TbSUMO could be activated by TbAos1/TbUba2 and conjugated to TbUba2 in the presence of ATP (Fig. 3a), implying again TbAos1/TbUba2 should be the E1 enzyme of the SUMOylation system.

E1-E2 interactions accelerate the SUMO transfer from E1 to a cysteine residue of E2 by forming a thioester bond, and E2 utilizes the energy stored in thioester bond to transfer SUMO to lysine ε-amino group of target protein directly or through E3 enzymes associated with Ubc9^23^. In order to investigate whether TbAos1/TbUba2 have the ability of transferring TbSUMO to TbUbc9, *in vitro* TbUbc9-SUMOylation assay was performed in the presence or absence of TbAos1 or TbUba2 (Fig. 3b). The results demonstrated that TbSUMO could be transferred from TbUba2 to TbUbc9, and the E1 holoenzyme was necessary for this process.

### Identification of the active sites of TbUba2 and TbUbc9

**Figure 4:**
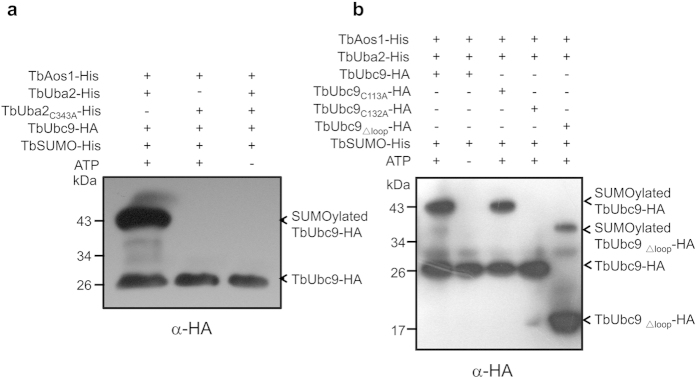
Identification of the active cysteine residues of TbUba2 and TbUbc9. (**a**) *In vitro* SUMOylation of TbUbc9-HA using TbUba2-His or TbUba2_C343A_-His in the presence or absence of ATP. The thioester linkage between TbUba2 and TbSUMO was located at C343 residue of TbUba2. (**b**) *in vitro* SUMOylation of TbUbc9-HA, TbUbc9_C113A_-HA, TbUbc9_C132A_-HA and TbUbc9_Δloop_-HA with or without ATP. It indicates that the thioester linkage was formed between C132 residue of TbUbc9-HA and the GG motif of TbSUMO. The samples were fractionated by SDS-PAGE (12%) and analyzed by immune blotting using anti-HA antibody.

Some conserved cysteine residues are critical for SUMO conjugation to Uba2 and Ubc9^6^,^7^. Sequence alignments indicated that TbUba2 and TbUbc9 contain the conserved active cysteine residues, Cys343 for TbUba2 and Cys132 for TbUbc9, respectively (see Supplementary Fig. S1a & S1c online). Thus, TbUba2-Cys343 and TbUbc9-Cys132 were mutated to Alanine for investigation of their significance. *In vitro* Ubc9-SUMOylation assays demonstrated that TbUba2-C343A mutant lost the ability to transfer TbSUMO to TbUbc9 (Fig. 4a), suggesting the essential role of Cys343 of TbUba2 in SUMO-conjugating process. Sequence analysis of TbUbc9 indicated that this protein contains another unconserved cysteine (Cys113) and an additional loop (TbUbc9_R31-S71_) (see Supplementary Fig. S1b online). In order to investigate whether TbUbc9-Cys113 and TbUbc9_R31-S71_ participate in E1-E2 SUMO transferring, *in vitro* TbUbc9-SUMOylation assays were performed with HA-tagged TbUbc9, TbUbc9_C132A,_ TbUbc9_C113A_ and TbUbc9_∆loop_ (Fig. 4b). The results showed that Cys132 is the active site of TbUbc9, and depletion of the extra loop of TbUbc9 did not affect TbSUMO transfer from E1 to E2.

### *In vitro* SUMOylation of TbCentrin3 by the SUMOylation system of *T. brucei*

**Figure 5:**
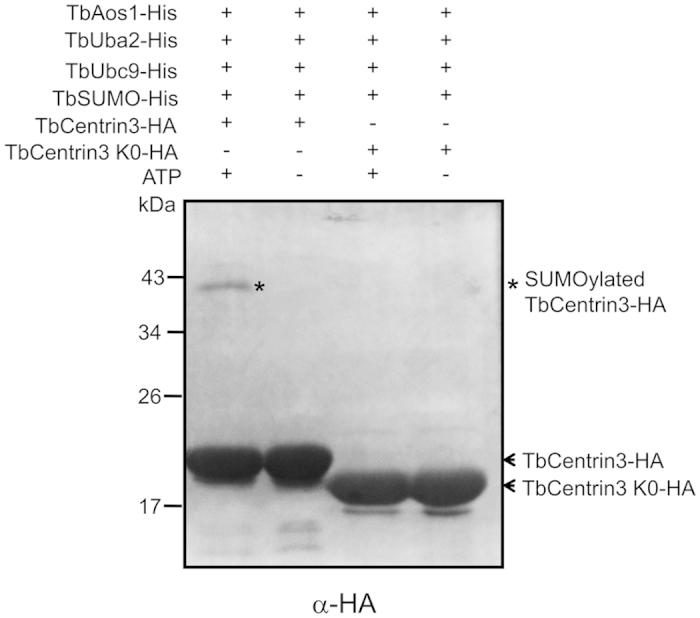
*In vitro* SUMOylation of TbCentrin3-HA and TbCentrin3 K0-HA in the presence or absence of ATP TbCentrin3 K0-HA was the mutant in which all the lysine residues were mutated to arginine except the deleted C-terminal lysine K164 and K165. The samples were fractionated by SDS-PAGE (12%) and analyzed by immune blotting using anti-HA antibody. SUMOylated Tbcentrin3-HA is indicated by asterisk (*).

It is well known that SUMO can be transferred to its targets by Ubc9 directly or with the assistance of E3 ligase. To investigate whether TbUbc9 is able to SUMOylate protein directly, *in vitro* SUMOylation of potential targets was performed. Previous studies showed that human centrin1 and centrin2 can be SUMOylated without E3 ligase *in vitro*^24^. Thus, a centrin homologue (TbCentrin3) of *T. brucei*, a flagellar protein associated with cell motility^25^, was supposed to be a target of SUMOylation. In order to confirm this, *In vitro* SUMOylation of TbCentrin3 was performed without E3 ligase. The result showed that TbCentrin3 could be SUMOylated by the reconstructed SUMOylation system, which suggested the ability of TbUbc9 to SUMOylate target. However, other potential targets of TbSUMO, such as aurora kinase homologue (TbAUK1) and RTF1 (Replication termination factor 1) homologue in *T. bruce*i, could not be SUMOylated *in vitro* by the reconstructed SUMOylation system (data not shown), suggesting E3 ligases might be required for some targets’ SUMOylation. Subsequently, we tried to identify which lysine residue of TbCentrin3 is responsible for SUMO conjugation. However, neither a single lysine nor multiple lysine residues mutation could block SUMOylation of TbCentrin3 (data not shown). Only the mutant wherein all lysine residues were replaced with arginine eliminated the SUMOylation of TbCentrin3 (Fig. 5). The lack of lysine specificity might be also due to the lack of E3 ligase in the reconstructed SUMOylation system.

## Discussion

Since the discoveries of ubiquitin and other ubiquitin-like proteins, it has been found that ubiquitin-like modifiers exist widely in eukaryotic organisms. In the last decades, SUMO has been extensively studied. It has been revealed that SUMO is involved in a number of recellular processes in higher organism, such as protein translocation, DNA pair, chromosome segregation, and so on^26^. However, studies of the SUMOylation in some early-branching ancient organisms, such as *Trypanosoma brucei*, are insufficient. Recently, our group reported the essential role of the SUMO homologue (TbSUMO) of *T. brucei* in cell life^20^, which suggests that TbSUMO may be multi-functional in *T. brucei*. In addition, proteomic analyses of SUMOylated proteins of *Trypanosoma cruzi*, a close relative species of *T. brucei*, further revealed diverse roles of the SUMO homologue in trypanosome^27^. Although some roles of SUMO may be conserved from *T. brucei* to human, studies of the SUMO system of *T. brucei* will promote our understanding on how cellular processes are regulated by SUMOylation.

In this study, we employed *in silico* analysis and biochemical methods to identify the E1 and E2 enzymes involved in SUMOylation in *T. brucei*. Sequence alignment revealed three proteins (TbAos1, TbUba2 and TbUbc9) in *T. brucei* share high similarity with human Aos1, Uba2 and Ubc9 respectively, implying they are homologues of E1 and E2. GST pull-down assay demonstrated the interactions between TbAos1, TbUba2, TbUbc9 and TbSUMO, indicating that TbAos1/TbUba2 and TbUbc9 are potential E1 and E2 enzymes of SUMOylation pathway. *In vitro* SUMOylation assay confirmed the activity of TbAos1/TbUba2 and TbUbc9 in SUMOylation pathway.

*In vitro* SUMOylation assay has been proved to be a useful tool to verify SUMO targets. Reconstruction of *in vitro* SUMOylation for *T. brucei* is essential for exploring functions of SUMOylation system in this organism. With the identification of E1 and E2 of the SUMO system of *T. brucei*, we recombinantly expressed and purified recombinant TbAos1, TbUba2, TbUbc9 and TbSUMO, and successfully established the *in vitro* SUMOylation system. Using the *in vitro* SUMOylation assay, the enzyme active sites of TbUba2 and TbUbc9 were identified to be located at Cys343 and Cys132, respectively. Subsequently, in this study, a centrin homologue in *T. brucei* (TbCentrin3) was identified to be a target of TbSUMO in the absence of E3 ligase. Meanwhile, this suggested that E1 and E2 enzymes are sufficient for SUMOylation of some proteins. TbCentrin3 has been reported to be localized to the flagellum and play an important role in cell motility and stabilization of inner-arm dynein complex^25^. SUMO-modified TbCentrin3 might be involved in a potential unrevealed biological process in *T. brucei*.

Although TbCentrin3 has been proved to be a SUMO target, we failed in identification of the lysine residue responsible for SUMOylation. Only the mutation that all lysine residues replaced with arginine can block the SUMOylation of TbCentrin3, which implies that SUMOylation of TbCentrin3 could occur at multiple sites, and might be tightly regulated by E3 ligase *in vivo*. Although *in vitro* SUMOylation assay can be used to identify some targets of SUMO in *T. brucei*, including E3 ligase in the assay may be essential for the identification of more targets of SUMO and specific SUMOylation sites of the targets. Thus, identification of E3 is necessary for understanding the cellular process in detail.

In summary, we identified TbAos1/TbUba2 and TbUbc9 to be the *bona fide* E1 and E2 ligases of the SUMOylation system of *T. brucei* and succeeded in reconstructing the SUMOylation system *in vitro* using these proteins. The *in vitro* SUMOylation system should be a useful tool for further study of SUMOylation in *T. brucei*.

## Materials and methods

### *In-silico* analysis

*In-silico* analysis was performed on webserver of TritrypDB database (http://tritrypdb.org/tritrypdb/). The primary sequences of Uba2, Aos1, and Ubc9 from different species (*Homo. sapience, Saccharomyces cerevisiae, Drosophila melanogaster*, and *Arabidopsis thaliana*) were blasted against the TritrypDB database respectively. Three potential homologues (Tb11.02.5410, Tb927.5.3430 and Tb927.2.2460 corresponding to TbAos1, TbUba2 and TbUbc9 respectively) were identified. The primary sequences of the potential homologues were subjected for domain analysis on SMART webserver (smart.embl-heidelberg.de). Sequence alignment was performed by using ClustalW2 (www.ebi.ac.uk/Tools/msa/clustalw2/).

### Gene cloning and plasmid construction

All genes were amplified from *T. brucei* genomic DNA by PCR. The sequence encoding residues 1–108 of TbSUMO was cloned into the pET28a(+) vector which contained opening reading frame (ORF) of 6 × His tag at the N-terminus. The genes encoding the subunits of E1 activating enzyme (TbAos1 and TbUba2 corresponding to Tb11.02.5410 and Tb927.5.3430 respectively) were cloned into pET22b(+) vector. The E2 conjugating enzyme TbUbc9, corresponding to Tb927.2.2460, was cloned into the pET22b (+) vector which contained ORF of 6 × His tag or hemagglutinin-tag (HA-tag). TbUba2 and TbUbc9 mutants were generated by the site-directed mutagenesis. Meanwhile, TbSUMO and TbAos1 fragments were cloned into the pGEX4T-1 vector for pull-down assay. TbCentrin3 were amplified by PCR and cloned into the pET22b(+) vector which contained ORF of HA-tag at the C-terminus.

### Site-directed mutagenesis and deletion mutagenesis

All the mutants were cloned into the pET22b(+) vector and contained HA-tag at the C-terminus. TbUba2_C343A_, TbUbc9_C113A_, TbUbc9_C132A_ were amplified by extension PCR using a pair of mutagenic primers. After PCR reaction, the products were digested with *Dpn*I (TaKaRa, Dalian, China) overnight at 37 °C. The plasmids were then transformed into *E. Coli* BL21 (DE3) for overexpression of proteins. As for the TbCentrin3 mutants, all the lysine residues (K27, K47, K59, K63, K90, K96, K110, K120, K136 and K153) were mutated to arginine, except the C-terminal K164 and K165 deletion mutant (TbCentrin3 K0), which was generated by multiple-site-directed mutagenesis step by step.

A loop of TbUbc9_R31-S71_ was deleted and the mutant TbUbc9_Δloop_ was generated by deletion mutagenesis using a pair of chimeric primers by overlap extension PCR procedure, then the product was digested by *Dpn*I at 37 °C and transformed into *E. Coli* BL21 (DE3).

### Recombinant protein expression and purification

All the plasmid constructs were transformed into *Escherichia coli* BL21 (DE3) respectively. The transformed cells were cultured in Luria-Bertani (LB) broth at 37 °C till OD_600_ = 0.8 and then induced with 0.5 mM IPTG. The expression of protein was carried out at 16 °C for 20 hours except for TbSUMO, which was expressed at 37 °C for 5 hours. The induced cells were harvested by centrifugation at 14000 g and resuspended in 20 mL lysis buffer (20 mM Tris, 500 mM NaCl, pH 7.6), then lysed by sonication. The lysate was centrifuged at 14000 g and 4 °C for 20 min to remove precipitate. The supernatant was loaded on Ni^2+^-NTA column (GE Health, chelating sepharose, 17-0575-02). The column was balanced with 20 mL lysis buffer and washed with 20 mL lysis buffer containing 50 mM imidazol. The recombinant proteins were eluted with lysis buffer containing 500 mM imidazol. The fractions containing the recombinant protein were collected and dialyzed against SUMOylation reaction buffer containing 20 mM Tris, 100 mM NaCl, 1 mM DTT and 1 mM EDTA at 4 °C. The mutants of TbUba2 and TbUbc9 were expressed and purified using the same method.

### GST pull-down assay

GST pull-down assay was employed to identify the interactions between the components of SUMOylation system. In brief, GST-fused proteins were purified as previously described and incubated with prepared glutathione sepharose beads (GE Health, Glutathione Sepharose 4B, 17-0756-01) on the rotating incubator at room temperature for 30 minutes, and then the beads were collected and washed 3 times. 0.1 mg/mL of input proteins were dissolved in the reaction buffer (20 mM Tris, 100 mM NaCl, 1 mM DTT and 1 mM EDTA) and incubated with the beads for 30 minutes. After removing the supernatant, the beads were washed with the reaction buffer four times. The target proteins were washed down with 10% SDS. These elutes were then analyzed and detected by SDS-PAGE and western blotting.

### *In vitro* SUMOylation assay

TbSUMO, TbAos1/TbUba2, and TbUbc9 were purified and dialyzed against the reaction buffer. Subsequently, 15 μg TbSUMO, 9 μg TbAos1, 3 μg TbUba2 and 20 μg TbUbc9 were mixed together to 50 μL volumes in the presence of 1 mM ATP and 2.5 mM Mg^2+^. The *in vitro* SUMOylation reaction was incubated at 27 °C for 2 hours and terminated with 2 × SDS-PAGE loading buffer. The samples were tested with 12% SDS-PAGE, and then analyzed by western blotting. The antibodies used in the western blotting analysis were His probe (sc-8036, Santa Cruz Biotechnology, mouse monoclonal antibody), HA probe (sc-7392, Santa Cruz Biotechnology, mouse monoclonal antibody against internal region of influenza hem agglutinin (HA) protein, used at 1:1000 dilution) and Goat-anti-mouse IgG-HRP (sc-2005, Santa Cruz Biotechnology, conjugated with HRP). Finally, the proteins were detected by luminescent image analyzer Image Quant LAS-4000 Mini (GE Healthcare).

## Author Contributions

K.Y., S.L. and X.T. designed the research. K.Y. and S.L. performed the experiments and data analysis. K.Y., S.L., X.Z., J.N. and X.T. wrote the paper.

## Additional Information

**How to cite this article**: Ye, K. *et al*. Identification of enzymes involved in SUMOylation in *Trypanosoma brucei*. *Sci. Rep*. **5**, 10097; doi: 10.1038/srep10097 (2015).

## Supplementary Material

Supplementary Information
