# Supplementary material for: Identification of enzymes involved in SUMOylation in Trypanosoma brucei
Source: Sci Rep. 2015 May 11;5:10097. doi: 10.1038/srep10097 (PMC4426598; doi:10.1038/srep10097)
Supplement: Supplementary Information [file srep10097-s1.pdf]

Supplementary Information

Identification of enzymes involved in SUMOylation in *Trypanosoma brucei*

KaiqinYe[1], Xuecheng Zhang[2,3], Jun Ni[1], Shanhui Liao[1,*] and Xiaoming Tu[1,*]

[1] Hefei National Laboratory for Physical Sciences at Microscale, School of Life Sciences, University of Science and Technology of China, Hefei, Anhui 230026, P.R. China

[2] School of Life Sciences, Anhui University, Hefei, Anhui 230039, P.R. China

[3]Anhui Provincial Engineering Technology Research Center of Microorganisms and Biocatalysis, 111 Jiulong Road, Hefei, Anhui 230601, P.R. China

[*]Correspondence to: Shanhui Liao, E-mail: ajsod@mail.ustc.edu.cn, and Xiaoming Tu, E-mail: xmtu@ustc.edu.cn

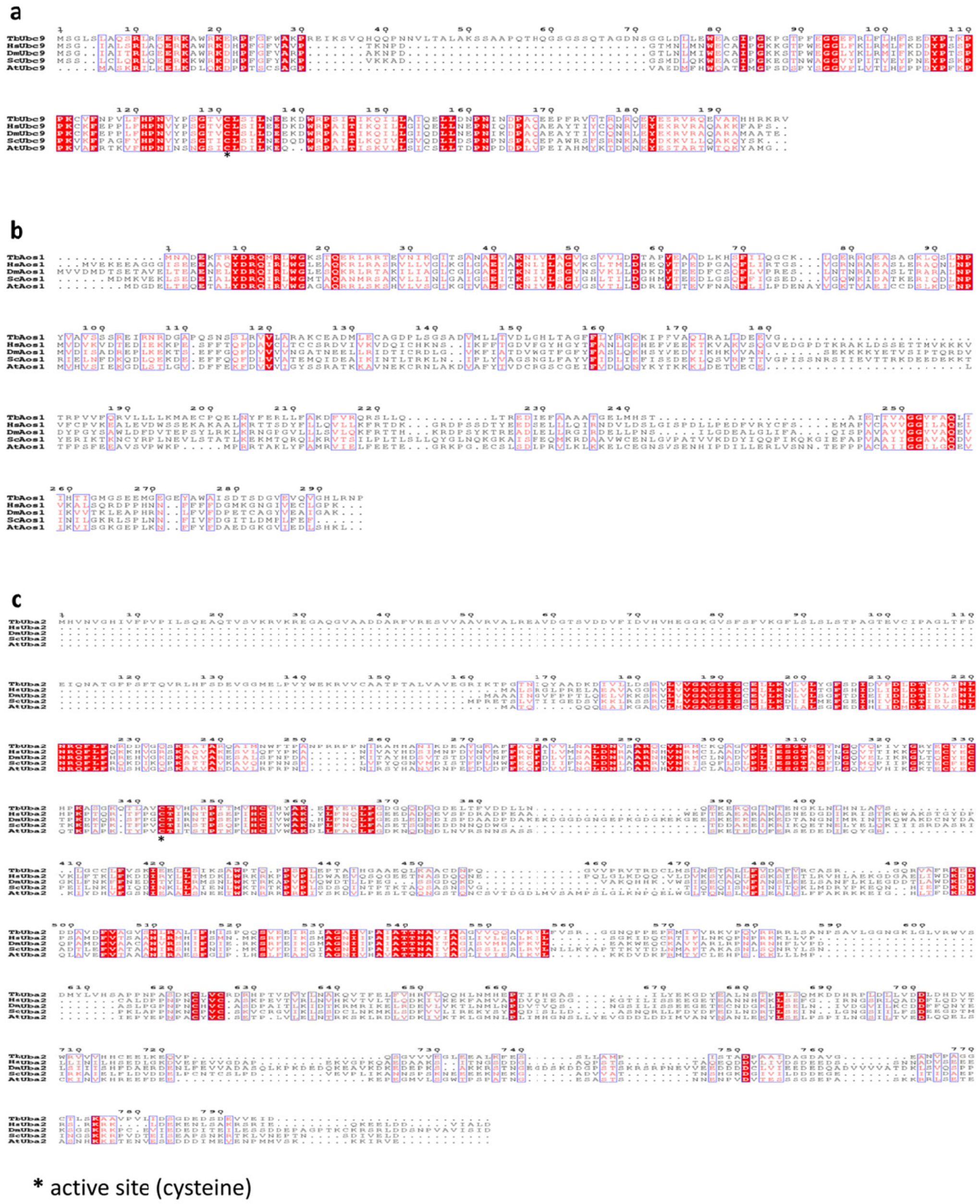

Figure S1: Sequence alignment of Ubc9 (a), Aos1 (b) and Uba2 (c) from *Trypanosoma brucei*, *Homo sapiens*, *Drosphila melanogaster*, *Saccharomyces cerevisiae* and *Arabidopsis thaliana.*
